# Supplementary material for: DVGfinder: A Metasearch Tool for Identifying Defective Viral Genomes in RNA-Seq Data
Source: Viruses. 2022 May 23;14(5):1114. doi: 10.3390/v14051114 (PMC9144107; doi:10.3390/v14051114)
Supplement: Supplementary file 1 [file viruses-14-01114-s001.zip › viruses-1731912-supplementary.pdf]

Text S1. Generation of the synthetic samples used for the evaluation

We generate two sets of synthetic samples, one based on the SARS-CoV-2 genome and the other based on the TuMV genome (both viral references are available in https://github.com/MJmaolu/DVGfinder/tree/v3/ExternalNeeds/references). For each group of samples (SARS-CoV-2 and TuMV) the list of random DVGs were generated once, then, the variable parameter was the total number of reads of the final fastq.

To generate each one of the synthetic datasets we followed the following steps:

1) Generate a list of random DVGs considering all the structural types (deletions forward, deletions reverse, insertions forward, insertions reverse, 5′ cb/sb and 3′ cb/sb) in equal proportions. While randomly generating each identifier, and before including it in the list, we confirmed that the DVG was not present in the previous set (neither in its direct nor in its reverse complementary form).
2) Generate the sequences corresponding to each DVG. These sequences are exactly the same as the reference except for the abnormal junction. For the reconstruction of the sequence, we followed the same rules that explained in Table 2 for calculating the theoretical length of the DVGs.
3) For each library size or number of total reads in the final fastq (N_{total}):
    a. Considering the number of total reads to be in the final fastq (N_{total}), the length of each concrete DVG ($length\ genome_i$), the length of the reads ($length\ reads$ = 100) and the proportion of the DVG in the sample ($proportion_i$), we calculated the number of synthetic reads ($N_{dvg\ i}$) to generate for each type of genome (equations 1-7 in S3).
    b. Given the DVG sequence, and the defined parameters, simulate the synthetic reads with the program wgsim [Wgsim (short read simulator) https://github.com/lh3/wgsim] without any error/mutation rate (parameters related to the addition of variation set to zero: -e0 -r0 -R0 -X0).
    c. Repeat points a and b for the complete viral genome.
    d. Concatenate all the reads in one fastq file.

In total, we generated 24 fastq samples, 12 based on each genome, with the library sizes (N_{total}) specified in Section 2.7. The composition ratio was 60% for the DVG population and 40% for the complete genome in all the datasets. About the composition of the DVG population, we used 216 different DVGs for the SARS-CoV-2 datasets and 72 for the TuMV ones in order to obtain the same density of defectives in both synthetic datasets (the length of the second is one third of the first so the number of defectives present is also one third).

Text S2. Calculation of the number of synthetic reads ($N_{dvg\ i}$) to generate for each one of the DVGs composing the synthetic datasets

We took as $proportion_i$ the ratio of the *genome*$_i$ in the sample, *i.e.*, in a sample composed by the complete virus and one type of DVG, a proportion of 0.5 means that for each complete genome are one DVG. Taking this into account, and knowing that the mean depth of a sample is defined by:

$$meanDepth = \frac{N \times length\ reads}{length\ genome} \quad (1),$$

and that the total number of reads in the final synthetic dataset is:

$$N_{total} = N_{wt} + N_{dvg_1} + N_{dvg_2} + \cdots + N_{dvg_n} = N_i + \cdots + N_n \quad (2).$$

We can extract the total number of reads (N_{total}) of Eq. 1 and substitute in Eq. 2, obtaining

$$N_{total} = \sum_{i=1}^{n} \frac{meanDepth_i \times length\ genome_i}{length\ reads} \quad (3).$$

Also, if all the genomes present in the sample share a common coordinate where the proportion of them can be calculated ($meanDepth_{total\ in\ Common\ Coordinate}$), considering as *genome*$_i$ proportion:

$$proportion_i = \frac{number\ genomes_i}{total\ genomes} = \frac{meanDepth_i}{meanDepth_{total\ in\ Common\ Coordinate}} \quad (4).$$

We can now write the mean depth of each genome ($meanDepth_i$) as a function of the common variable $meanDepth_{total\ in\ Common\ Coordinate}$:

$$N_{total} = \frac{meanDepth_{total\ in\ Common\ Coordinate}}{length\ reads} \times \sum_{i=1}^{n} (proportion_i \times length\ genome_i) \quad (5)$$

and

$$meanDepth_{total\ in\ Common\ Coordinate} = \frac{N_{total} \times length\ reads}{\sum_{i=1}^{n} (proportion_i \times length\ genome_i)} \quad (6).$$

Knowing the average depth in the common coordinate, it is finally possible to calculate the number of reads ($N_{dvg\ i}$) with which each genome must be generated in the synthetic sample in order to maintain the desired ratio:

$$N_{dvg\ i} = \frac{proportion_i \times meanDepth_{total\ in\ Common\ Coordinate} \times length\ genome_i}{length\ reads} \quad (7).$$

Figure S1. A recommended general pipeline for real samples

(* DI-tector recommendation)

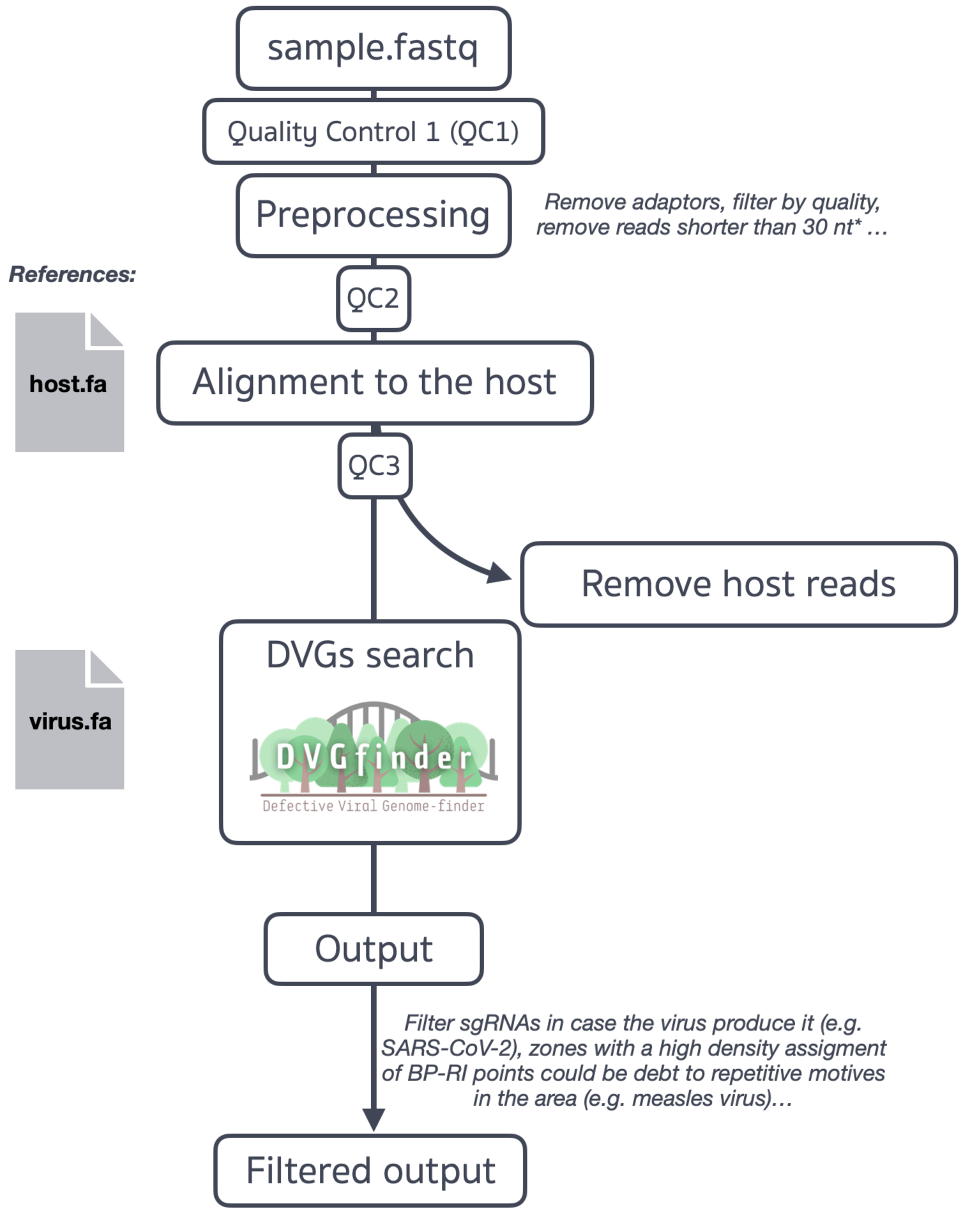

Figure S2. Example of the behavior of the two search algorithms on the SARS-CoV-2 small (100 K reads) and large (10 M reads) synthetic samples. For simplicity, the DVG sense has not been considered. Red points represent the true events in the sample while the crosses represent candidates identified by each program (A-B: ViReMa-a; C-D: DI-tector). True positives correspond to cases in which red points and crosses coincide, orphan crosses correspond to false positives and orphan red points to false negatives. ViReMa-a works better than DI-tector at both coverages.

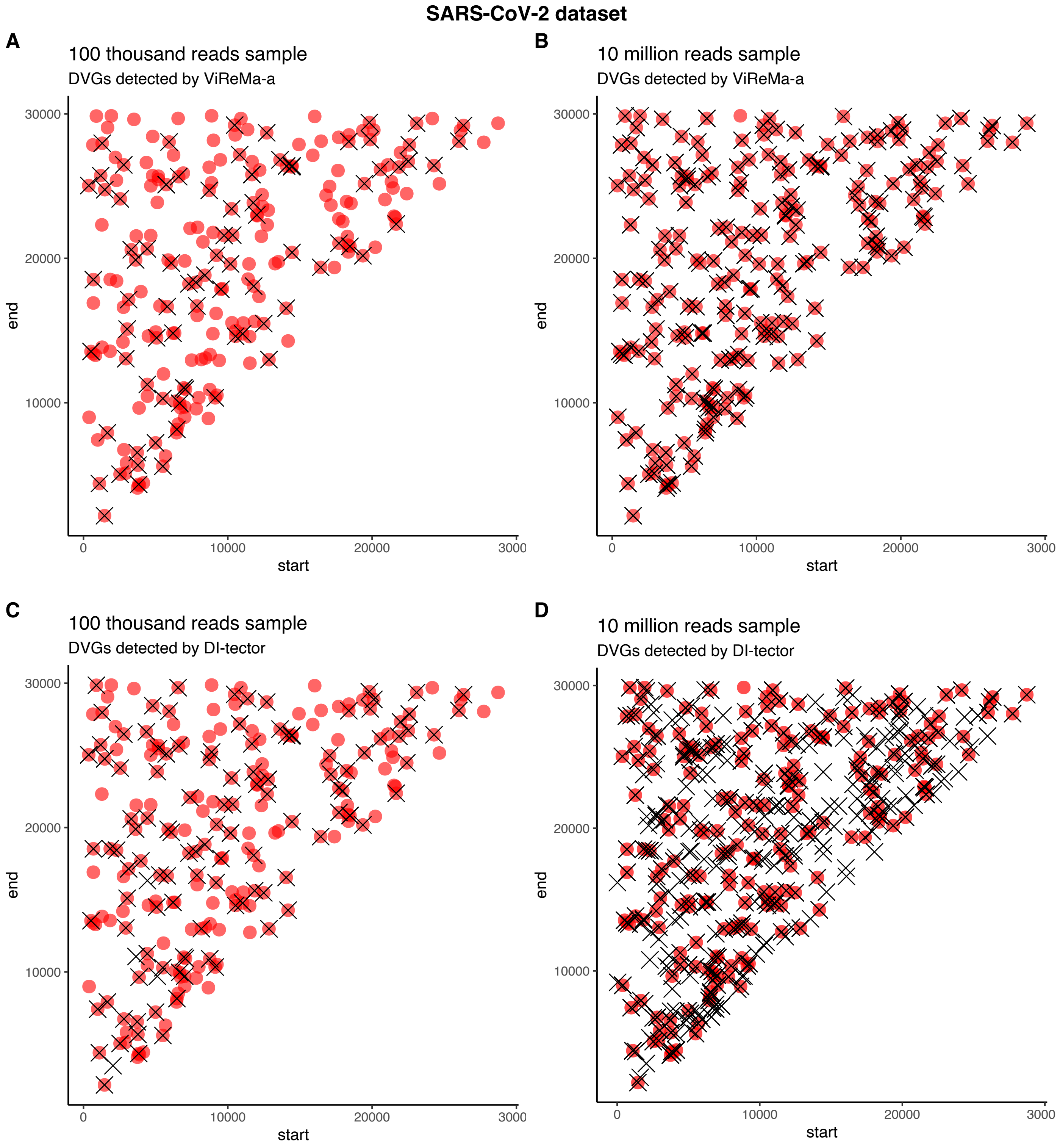

Figure S3. Copybacks detected by DVGfinder (black points) in samples SRR8719995 (first column), SRR8719996 (center column) and SRR8719998 (right column). The red asteriscs are the cb validated by RT-PCR by the authors of the samples. The output is separated by program of detection (ViReMa-a: *a-c*, DI-tector: *d-f*) and by DVGfinder mode (Metasearch: *g-i*, Consensus: *j-l* and Filtered: *m-o*). The size of points is proportional to the number of reads found for each case.

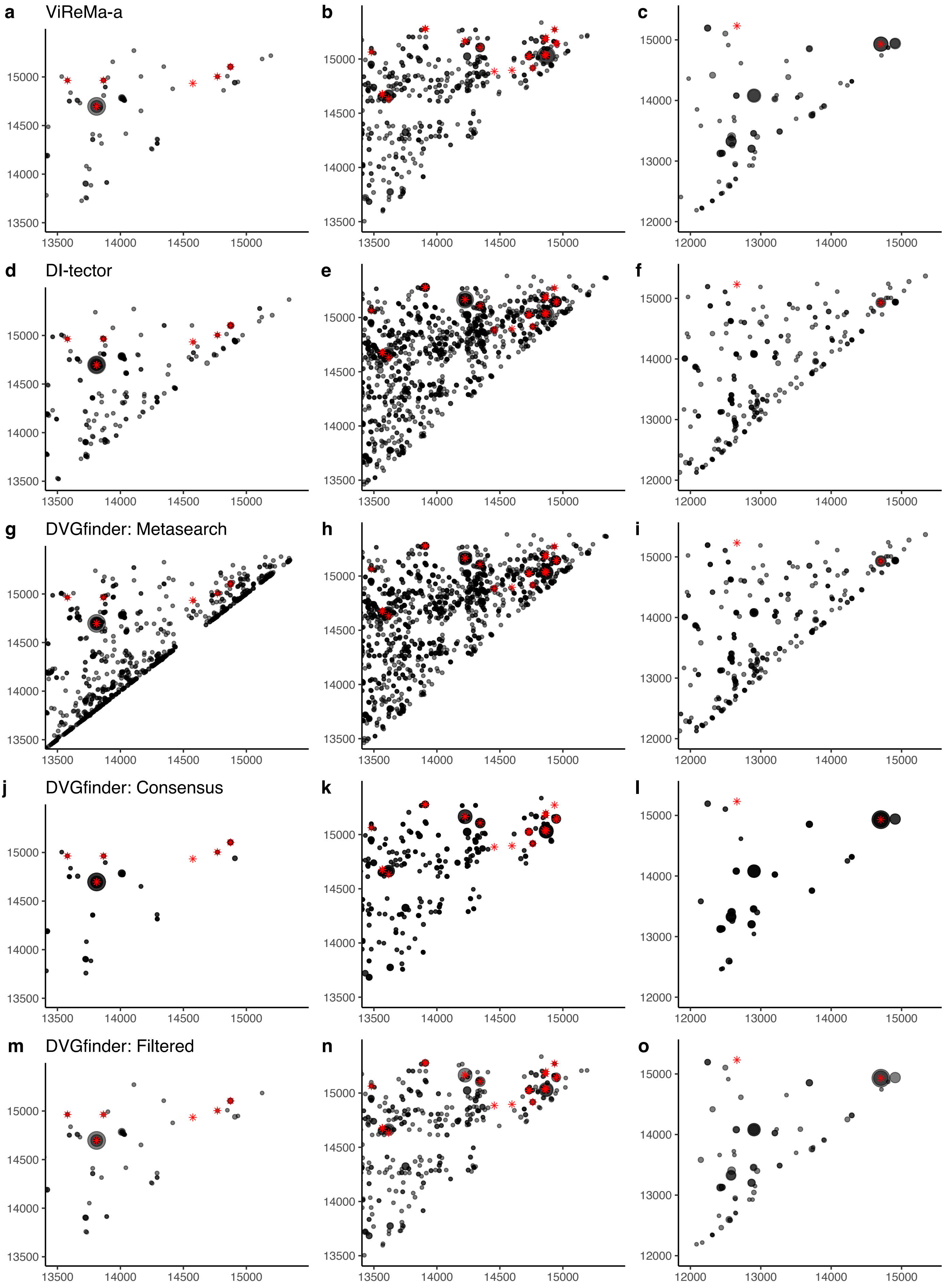
a
ViReMa-a
b
c
d
DI-tector
e
f
g
DVGfinder: Metasearch
h
i
j
DVGfinder: Consensus
k
l
m
DVGfinder: Filtered
n
o
13500
14000
14500
15000
12000
13000

Table S1. List of random DVGs generated for each synthetic dataset. Also the proportion of each genome is indicated.

The list of the random DVGs present in the synthetic datasets and its ratio is available in [45]. Also, the 24 fastq files used for the evaluation can be download [46].

SARS-CoV-2 dataset:

cID_DI	sense	BP	RI	DVG_type	proportion	length_dvg
++_8276_21139	++	8276	21139	Deletion_forward	0.003	17041
++_10338_21607	++	10338	21607	Deletion_forward	0.003	18635
++_650_27853	++	650	27853	Deletion_forward	0.003	2701
++_7019_19818	++	7019	19818	Deletion_forward	0.003	17105
++_984_7423	++	984	7423	Deletion_forward	0.003	23465
++_17055_24978	++	17055	24978	Deletion_forward	0.003	21981
++_8165_12993	++	8165	12993	Deletion_forward	0.003	25076
++_22400_24481	++	22400	24481	Deletion_forward	0.003	27823
++_26334_29200	++	26334	29200	Deletion_forward	0.003	27038
++_19865_28219	++	19865	28219	Deletion_forward	0.003	21550
++_1930_29865	++	1930	29865	Deletion_forward	0.003	1969
++_24659_25162	++	24659	25162	Deletion_forward	0.003	29401
++_7494_12948	++	7494	12948	Deletion_forward	0.003	24450
++_21970_27319	++	21970	27319	Deletion_forward	0.003	24555
++_18227_23933	++	18227	23933	Deletion_forward	0.003	24198
++_3025_15101	++	3025	15101	Deletion_forward	0.003	17828
++_11858_15625	++	11858	15625	Deletion_forward	0.003	26137
++_9005_28173	++	9005	28173	Deletion_forward	0.003	10736
++_1665_29052	++	1665	29052	Deletion_forward	0.003	2517
++_8710_26306	++	8710	26306	Deletion_forward	0.003	12308
++_682_16908	++	682	16908	Deletion_forward	0.003	13678
++_3612_19888	++	3612	19888	Deletion_forward	0.003	13628
++_5185_25687	++	5185	25687	Deletion_forward	0.003	9402
++_11512_14577	++	11512	14577	Deletion_forward	0.003	26839
++_10513_28550	++	10513	28550	Deletion_forward	0.003	11867
++_13620_26819	++	13620	26819	Deletion_forward	0.003	16705
++_18535_23805	++	18535	23805	Deletion_forward	0.003	24634
++_1100_4403	++	1100	4403	Deletion_forward	0.003	26601
++_10553_14994	++	10553	14994	Deletion_forward	0.003	25463
++_6203_14816	++	6203	14816	Deletion_forward	0.003	21291
++_8964_14780	++	8964	14780	Deletion_forward	0.003	24088
++_11073_15523	++	11073	15523	Deletion_forward	0.003	25454
++_19465_25174	++	19465	25174	Deletion_forward	0.003	24195
++_4417_20657	++	4417	20657	Deletion_forward	0.003	13664
++_20890_24069	++	20890	24069	Deletion_forward	0.003	26725
++_11531_12740	++	11531	12740	Deletion_forward	0.003	28695
++_24869_21446	++	24869	21446	Insertion_forward	0.003	33327
++_10454_4429	++	10454	4429	Insertion_forward	0.003	35929
++_13843_1289	++	13843	1289	Insertion_forward	0.003	42458
++_18827_8410	++	18827	8410	Insertion_forward	0.003	40321
++_18431_2292	++	18431	2292	Insertion_forward	0.003	46043
++_29406_19805	++	29406	19805	Insertion_forward	0.003	39505
++_28837_19357	++	28837	19357	Insertion_forward	0.003	39384
++_26467_21081	++	26467	21081	Insertion_forward	0.003	35290
++_11264_4420	++	11264	4420	Insertion_forward	0.003	36748
++_21529_12327	++	21529	12327	Insertion_forward	0.003	39106
++_22318_1271	++	22318	1271	Insertion_forward	0.003	50951
++_29624_3496	++	29624	3496	Insertion_forward	0.003	56032
++_22735_17696	++	22735	17696	Insertion_forward	0.003	34943

++_22373_21666	++	22373	21666	Insertion_forward	0.003	30611
++_23851_11766	++	23851	11766	Insertion_forward	0.003	41989
++_23688_17146	++	23688	17146	Insertion_forward	0.003	36446
++_13093_8452	++	13093	8452	Insertion_forward	0.003	34545
++_14771_11028	++	14771	11028	Insertion_forward	0.003	33647
++_26425_24290	++	26425	24290	Insertion_forward	0.003	32039
++_25728_1190	++	25728	1190	Insertion_forward	0.003	54442
++_25403_2283	++	25403	2283	Insertion_forward	0.003	53024
++_28440_4797	++	28440	4797	Insertion_forward	0.003	53547
++_15537_10290	++	15537	10290	Insertion_forward	0.003	35151
++_20211_9217	++	20211	9217	Insertion_forward	0.003	40898
++_9702_7012	++	9702	7012	Insertion_forward	0.003	32594
++_7921_6449	++	7921	6449	Insertion_forward	0.003	31376
++_23871_5113	++	23871	5113	Insertion_forward	0.003	48662
++_13566_1842	++	13566	1842	Insertion_forward	0.003	41628
++_26320_21854	++	26320	21854	Insertion_forward	0.003	34370
++_19612_11469	++	19612	11469	Insertion_forward	0.003	38047
++_17895_9617	++	17895	9617	Insertion_forward	0.003	38182
++_14895_5002	++	14895	5002	Insertion_forward	0.003	39797
++_5674_3770	++	5674	3770	Insertion_forward	0.003	31808
++_8520_6566	++	8520	6566	Insertion_forward	0.003	31858
++_22849_21599	++	22849	21599	Insertion_forward	0.003	31154
++_25463_5190	++	25463	5190	Insertion_forward	0.003	50177
--_26631_4356	--	26631	4356	Deletion_reverse	0.003	7629
--_7920_1655	--	7920	1655	Deletion_reverse	0.003	23639
--_16615_2767	--	16615	2767	Deletion_reverse	0.003	16056
--_18335_7778	--	18335	7778	Deletion_reverse	0.003	19347
--_24406_12379	--	24406	12379	Deletion_reverse	0.003	17877
--_26335_14425	--	26335	14425	Deletion_reverse	0.003	17994
--_10515_9246	--	10515	9246	Deletion_reverse	0.003	28635
--_22077_7387	--	22077	7387	Deletion_reverse	0.003	15214
--_22318_12730	--	22318	12730	Deletion_reverse	0.003	20316
--_5600_5498	--	5600	5498	Deletion_reverse	0.003	29802
--_29223_10474	--	29223	10474	Deletion_reverse	0.003	11155
--_27968_1294	--	27968	1294	Deletion_reverse	0.003	3230
--_26393_14073	--	26393	14073	Deletion_reverse	0.003	17584
--_21789_8977	--	21789	8977	Deletion_reverse	0.003	17092
--_25037_363	--	25037	363	Deletion_reverse	0.003	5230
--_24704_8713	--	24704	8713	Deletion_reverse	0.003	13913
--_13049_2932	--	13049	2932	Deletion_reverse	0.003	19787
--_4445_4148	--	4445	4148	Deletion_reverse	0.003	29607
--_25800_11671	--	25800	11671	Deletion_reverse	0.003	15775
--_13548_521	--	13548	521	Deletion_reverse	0.003	16877
--_14492_5067	--	14492	5067	Deletion_reverse	0.003	20479
--_24119_2545	--	24119	2545	Deletion_reverse	0.003	8330
--_21518_18285	--	21518	18285	Deletion_reverse	0.003	26671
--_27884_14929	--	27884	14929	Deletion_reverse	0.003	16949
--_17853_9521	--	17853	9521	Deletion_reverse	0.003	21572
--_28382_17683	--	28382	17683	Deletion_reverse	0.003	19205
--_25723_4790	--	25723	4790	Deletion_reverse	0.003	8971
--_9655_6728	--	9655	6728	Deletion_reverse	0.003	26977
--_4273_3881	--	4273	3881	Deletion_reverse	0.003	29512
--_19369_17388	--	19369	17388	Deletion_reverse	0.003	27923
--_9966_6662	--	9966	6662	Deletion_reverse	0.003	26600
--_5047_2535	--	5047	2535	Deletion_reverse	0.003	27392
--_29681_10910	--	29681	10910	Deletion_reverse	0.003	11133

--_21579_9746	--	21579	9746	Deletion_reverse	0.003	18071
--_20911_18330	--	20911	18330	Deletion_reverse	0.003	27323
--_23001_12007	--	23001	12007	Deletion_reverse	0.003	18910
--_12695_28693	--	12695	28693	Insertion_reverse	0.003	45902
--_3826_4349	--	3826	4349	Insertion_reverse	0.003	30427
--_6596_25655	--	6596	25655	Insertion_reverse	0.003	48963
--_6952_11025	--	6952	11025	Insertion_reverse	0.003	33977
--_7991_10354	--	7991	10354	Insertion_reverse	0.003	32267
--_16810_24366	--	16810	24366	Insertion_reverse	0.003	37460
--_5298_16704	--	5298	16704	Insertion_reverse	0.003	41310
--_11373_28920	--	11373	28920	Insertion_reverse	0.003	47451
--_6315_14823	--	6315	14823	Insertion_reverse	0.003	38412
--_2734_14205	--	2734	14205	Insertion_reverse	0.003	41375
--_5802_16654	--	5802	16654	Insertion_reverse	0.003	40756
--_9401_12926	--	9401	12926	Insertion_reverse	0.003	33429
--_8750_10907	--	8750	10907	Insertion_reverse	0.003	32061
--_10540_14600	--	10540	14600	Insertion_reverse	0.003	33964
--_10262_23430	--	10262	23430	Insertion_reverse	0.003	43072
--_4666_25023	--	4666	25023	Insertion_reverse	0.003	50261
--_12038_22951	--	12038	22951	Insertion_reverse	0.003	40817
--_15888_27136	--	15888	27136	Insertion_reverse	0.003	41152
--_2800_6742	--	2800	6742	Insertion_reverse	0.003	33846
--_2977_5822	--	2977	5822	Insertion_reverse	0.003	32749
--_8878_25230	--	8878	25230	Insertion_reverse	0.003	46256
--_6399_10107	--	6399	10107	Insertion_reverse	0.003	33612
--_12210_26116	--	12210	26116	Insertion_reverse	0.003	43810
--_16434_19373	--	16434	19373	Insertion_reverse	0.003	32843
--_4658_21577	--	4658	21577	Insertion_reverse	0.003	46823
--_12162_17363	--	12162	17363	Insertion_reverse	0.003	35105
--_1455_2174	--	1455	2174	Insertion_reverse	0.003	30623
--_3852_9636	--	3852	9636	Insertion_reverse	0.003	35688
--_24171_29682	--	24171	29682	Insertion_reverse	0.003	35415
--_18354_20443	--	18354	20443	Insertion_reverse	0.003	31993
--_27736_28035	--	27736	28035	Insertion_reverse	0.003	30203
--_3309_20592	--	3309	20592	Insertion_reverse	0.003	47187
--_14455_26405	--	14455	26405	Insertion_reverse	0.003	41854
--_28728_29356	--	28728	29356	Insertion_reverse	0.003	30532
--_9482_26819	--	9482	26819	Insertion_reverse	0.003	47241
--_5930_28056	--	5930	28056	Insertion_reverse	0.003	52030
+-_12403_23593	+-	12403	23593	5cb/sb	0.003	35996
+-_11793_18074	+-	11793	18074	5cb/sb	0.003	29867
+-_21046_17703	+-	21046	17703	5cb/sb	0.003	38749
+-_26122_28866	+-	26122	28866	5cb/sb	0.003	54988
+-_25901_6915	+-	25901	6915	5cb/sb	0.003	32816
+-_18526_686	+-	18526	686	5cb/sb	0.003	19212
+-_20408_14450	+-	20408	14450	5cb/sb	0.003	34858
+-_5115_2905	+-	5115	2905	5cb/sb	0.003	8020
+-_18397_28546	+-	18397	28546	5cb/sb	0.003	46943
+-_16542_14044	+-	16542	14044	5cb/sb	0.003	30586
+-_9581_7812	+-	9581	7812	5cb/sb	0.003	17393
+-_20578_26144	+-	20578	26144	5cb/sb	0.003	46722
+-_10937_7066	+-	10937	7066	5cb/sb	0.003	18003
+-_26696_11682	+-	26696	11682	5cb/sb	0.003	38378
+-_17634_26079	+-	17634	26079	5cb/sb	0.003	43713
+-_20219_20768	+-	20219	20768	5cb/sb	0.003	40987
+-_8154_6472	+-	8154	6472	5cb/sb	0.003	14626

+-_27183_10819	+-	27183	10819	5cb/sb	0.003	38002
+-_22545_17969	+-	22545	17969	5cb/sb	0.003	40514
+-_26472_2791	+-	26472	2791	5cb/sb	0.003	29263
+-_10291_5498	+-	10291	5498	5cb/sb	0.003	15789
+-_23084_29353	+-	23084	29353	5cb/sb	0.003	52437
+-_29853_892	+-	29853	892	5cb/sb	0.003	30745
+-_8908_8652	+-	8908	8652	5cb/sb	0.003	17560
+-_5684_6307	+-	5684	6307	5cb/sb	0.003	11991
+-_16465_28111	+-	16465	28111	5cb/sb	0.003	44576
+-_13335_8766	+-	13335	8766	5cb/sb	0.003	22101
+-_6547_3723	+-	6547	3723	5cb/sb	0.003	10270
+-_29687_6557	+-	29687	6557	5cb/sb	0.003	36244
+-_12851_12986	+-	12851	12986	5cb/sb	0.003	25837
+-_5731_25105	+-	5731	25105	5cb/sb	0.003	30836
+-_4990_7217	+-	4990	7217	5cb/sb	0.003	12207
+-_26016_28109	+-	26016	28109	5cb/sb	0.003	54125
+-_20832_18464	+-	20832	18464	5cb/sb	0.003	39296
+-_17141_3129	+-	17141	3129	5cb/sb	0.003	20270
+-_1475_24753	+-	1475	24753	5cb/sb	0.003	26228
-+_19674_6080	-+	19674	6080	3cb/sb	0.003	34054
-+_22918_21516	-+	22918	21516	3cb/sb	0.003	15374
-+_19762_13507	-+	19762	13507	3cb/sb	0.003	26539
-+_8997_7015	-+	8997	7015	3cb/sb	0.003	43796
-+_19359_20180	-+	19359	20180	3cb/sb	0.003	20269
-+_19615_10189	-+	19615	10189	3cb/sb	0.003	30004
-+_7873_16046	-+	7873	16046	3cb/sb	0.003	35889
-+_9190_16184	-+	9190	16184	3cb/sb	0.003	34434
-+_829_13289	-+	829	13289	3cb/sb	0.003	45690
-+_23334_12800	-+	23334	12800	3cb/sb	0.003	23674
-+_9125_10339	-+	9125	10339	3cb/sb	0.003	40344
-+_28876_20118	-+	28876	20118	3cb/sb	0.003	10814
-+_7855_16702	-+	7855	16702	3cb/sb	0.003	35251
-+_7308_18235	-+	7308	18235	3cb/sb	0.003	34265
-+_8989_387	-+	8989	387	3cb/sb	0.003	50432
-+_18559_11406	-+	18559	11406	3cb/sb	0.003	29843
-+_7904_22146	-+	7904	22146	3cb/sb	0.003	29758
-+_27856_22579	-+	27856	22579	3cb/sb	0.003	9373
-+_16022_29826	-+	16022	29826	3cb/sb	0.003	13960
-+_5540_11993	-+	5540	11993	3cb/sb	0.003	42275
-+_14176_14275	-+	14176	14275	3cb/sb	0.003	31357
-+_27152_6243	-+	27152	6243	3cb/sb	0.003	26413
-+_1852_18533	-+	1852	18533	3cb/sb	0.003	39423
-+_28090_18385	-+	28090	18385	3cb/sb	0.003	13333
-+_19631_13276	-+	19631	13276	3cb/sb	0.003	26901
-+_5869_19878	-+	5869	19878	3cb/sb	0.003	34061
-+_15481_12490	-+	15481	12490	3cb/sb	0.003	31837
-+_29870_8878	-+	29870	8878	3cb/sb	0.003	21060
-+_21338_25306	-+	21338	25306	3cb/sb	0.003	13164
-+_26986_2210	-+	26986	2210	3cb/sb	0.003	30612
-+_4495_14621	-+	4495	14621	3cb/sb	0.003	40692
-+_13384_677	-+	13384	677	3cb/sb	0.003	45747
-+_3976_17690	-+	3976	17690	3cb/sb	0.003	38142
-+_21548_3654	-+	21548	3654	3cb/sb	0.003	34606
-+_3737_4096	-+	3737	4096	3cb/sb	0.003	51975
-+_22508_26718	-+	22508	26718	3cb/sb	0.003	10582

TuMV dataset:

cID_DI	sense	BP	RI	DVG_type	proportion	length_dvg
++_5016_8357	++	5016	8357	Deletion_forward	0.003	6492
++_4533_4797	++	4533	4797	Deletion_forward	0.003	9569
++_2365_3647	++	2365	3647	Deletion_forward	0.003	8551
++_4022_7019	++	4022	7019	Deletion_forward	0.003	6836
++_1784_6060	++	1784	6060	Deletion_forward	0.003	5557
++_8649_9348	++	8649	9348	Deletion_forward	0.003	9134
++_4166_6105	++	4166	6105	Deletion_forward	0.003	7894
++_7365_9680	++	7365	9680	Deletion_forward	0.003	7518
++_2963_6925	++	2963	6925	Deletion_forward	0.003	5871
++_6000_6040	++	6000	6040	Deletion_forward	0.003	9793
++_55_8684	++	55	8684	Deletion_forward	0.003	1204
++_745_6507	++	745	6507	Deletion_forward	0.003	4071
++_4275_1502	++	4275	1502	Insertion_forward	0.003	12606
++_7574_5084	++	7574	5084	Insertion_forward	0.003	12323
++_8581_1436	++	8581	1436	Insertion_forward	0.003	16978
++_7321_1004	++	7321	1004	Insertion_forward	0.003	16150
++_8076_3193	++	8076	3193	Insertion_forward	0.003	14716
++_4845_3612	++	4845	3612	Insertion_forward	0.003	11066
++_5215_4600	++	5215	4600	Insertion_forward	0.003	10448
++_3792_3598	++	3792	3598	Insertion_forward	0.003	10027
++_7724_6110	++	7724	6110	Insertion_forward	0.003	11447
++_4061_2927	++	4061	2927	Insertion_forward	0.003	10967
++_8223_2746	++	8223	2746	Insertion_forward	0.003	15310
++_8365_7470	++	8365	7470	Insertion_forward	0.003	10728
--_6225_704	--	6225	704	Deletion_reverse	0.003	4312
--_8665_4479	--	8665	4479	Deletion_reverse	0.003	5647
--_7242_3859	--	7242	3859	Deletion_reverse	0.003	6450
--_5147_3516	--	5147	3516	Deletion_reverse	0.003	8202
--_6092_49	--	6092	49	Deletion_reverse	0.003	3790
--_2957_621	--	2957	621	Deletion_reverse	0.003	7497
--_4654_4560	--	4654	4560	Deletion_reverse	0.003	9739
--_8273_1909	--	8273	1909	Deletion_reverse	0.003	3469
--_2302_1051	--	2302	1051	Deletion_reverse	0.003	8582
--_9642_5893	--	9642	5893	Deletion_reverse	0.003	6084
--_8254_3000	--	8254	3000	Deletion_reverse	0.003	4579
--_8595_5065	--	8595	5065	Deletion_reverse	0.003	6303
--_7501_8757	--	7501	8757	Insertion_reverse	0.003	11089
--_918_8831	--	918	8831	Insertion_reverse	0.003	17746
--_8925_9229	--	8925	9229	Insertion_reverse	0.003	10137
--_1803_5741	--	1803	5741	Insertion_reverse	0.003	13771
--_5149_8484	--	5149	8484	Insertion_reverse	0.003	13168
--_552_5442	--	552	5442	Insertion_reverse	0.003	14723
--_6901_8812	--	6901	8812	Insertion_reverse	0.003	11744
--_2983_7883	--	2983	7883	Insertion_reverse	0.003	14733
--_176_490	--	176	490	Insertion_reverse	0.003	10147
--_1072_1974	--	1072	1974	Insertion_reverse	0.003	10735
--_3885_7826	--	3885	7826	Insertion_reverse	0.003	13774
--_3087_8779	--	3087	8779	Insertion_reverse	0.003	15525
+-_762_8125	+-	762	8125	5cb/sb	0.003	8887
+-_7968_6287	+-	7968	6287	5cb/sb	0.003	14255
+-_3458_7776	+-	3458	7776	5cb/sb	0.003	11234
+-_9776_9177	+-	9776	9177	5cb/sb	0.003	18953
+-_1706_7656	+-	1706	7656	5cb/sb	0.003	9362
+-_2677_3537	+-	2677	3537	5cb/sb	0.003	6214
+-_5359_7710	+-	5359	7710	5cb/sb	0.003	13069

+-_3497_970	+-	3497	970	5cb/sb	0.003	4467
+-_163_4932	+-	163	4932	5cb/sb	0.003	5095
+-_1039_8979	+-	1039	8979	5cb/sb	0.003	10018
+-_4015_102	+-	4015	102	5cb/sb	0.003	4117
+-_1460_2998	+-	1460	2998	5cb/sb	0.003	4458
-+_2354_4290	-+	2354	4290	3cb/sb	0.003	13022
-+_2387_1501	-+	2387	1501	3cb/sb	0.003	15778
-+_9114_6940	-+	9114	6940	3cb/sb	0.003	3612
-+_4967_8432	-+	4967	8432	3cb/sb	0.003	6267
-+_2543_1035	-+	2543	1035	3cb/sb	0.003	16088
-+_8434_7304	-+	8434	7304	3cb/sb	0.003	3928
-+_3409_4133	-+	3409	4133	3cb/sb	0.003	12124
-+_9054_3200	-+	9054	3200	3cb/sb	0.003	7412
-+_3324_6240	-+	3324	6240	3cb/sb	0.003	10102
-+_6126_9614	-+	6126	9614	3cb/sb	0.003	3926
-+_8306_7679	-+	8306	7679	3cb/sb	0.003	3681
-+_4255_7271	-+	4255	7271	3cb/sb	0.003	8140
